# Microwave Ablation Versus Nipple Sparing Mastectomy for Breast Cancer ≤5 cm: A Pilot Cohort Study

**DOI:** 10.3389/fonc.2020.546883

**Published:** 2020-10-07

**Authors:** Jie Yu, Zhi-yu Han, Ting Li, Wen-zhe Feng, Xiao-ling Yu, Yan-chun Luo, Han Wu, Jian Jiang, Jian-dong Wang, Ping Liang

**Affiliations:** ^1^ Department of Interventional Ultrasound, Chinese PLA General Hospital, Beijing, China; ^2^ Department of Ultrasound, People’s Hospital of Sanya, Sanya, China; ^3^ Department of Laboratory Medicine, The Fourth Hospital of Baotou City, Baotou, China; ^4^ Department of Ultrasound, Qingdao Municipal Hospital, Qingdao, China; ^5^ Department of General Surgery, Chinese PLA General Hospital, Beijing, China

**Keywords:** breast cancer, nipple, breast-sparing surgery, microwaves, ablation techniques

## Abstract

**Objectives:**

Compared with nipple sparing mastectomy (NSM), microwave ablation (MWA) is one relatively new modality indicated for selected breast cancer with nipple sparing and with little of evidence-based medical research for decision-making. The objective of this study was to compare the effect of ultrasound-guided percutaneous MWA and NSM for breast cancer.

**Materials and Methods:**

A retrospective cohort study was conducted in a single institution from 2014 to 2020. Women with invasive ductal carcinoma of the breast ≤ 5cm treated by MWA or NSM were enrolled. The primary end point was tumor progression and secondary end points included survival, cosmetic results, and complications.

**Results:**

21 patients in the MWA group and 43 in the NSM group were evaluated. The mean tumor size was 2.3 cm (range, 0.3–5.0 cm). Median follow-up was 26.7 months (range, 14.6–62.5 months). The mean age of MWA was 24 years older than that of the NSM group. All the patients achieved technique effectiveness. One local tumor progression and one ipsilateral breast recurrence occurred at 42 and 28 months after MWA, respectively. One ipsilateral breast recurrence and two bone metastasis occurred at 31.2, 34, and 30.5 months after NSM. Two groups had no significant difference in tumor progression (P = 0.16). No participants in both groups developed cancer related death (P > 0.99) and major complications (P > 0.99). However, MWA needed less hospitalization time (P < 0.001) and achieved better cosmetic results (P < 0.001).

**Conclusions:**

MWA achieved similar short term effect for breast cancer control and better cosmetic satisfaction compared with NSM in selected patients. MWA provides appropriate option for elderly patients who are unfit for surgery.

## Highlights

- As techniques with nipple sparing, microwave ablation achieved satisfactory effect in treating breast tumors ≤5 cm compared with nipple sparing mastectomy during a 26.7-month follow-up.
- Microwave ablation achieved better cosmetic satisfaction compared with nipple sparing mastectomy.
- MWA could be considered as an alternative minimally invasive treatment in early stage tumors and in the elderly cases considered unfit for surgery.

## Introduction

Among females, breast cancer (BC) is the most commonly diagnosed cancer (24.2% of the total cancer cases) and the leading cause of cancer death (15.0% of the total cancer deaths) worldwide (1). Surgical management of BC has undergone a dramatic evolution over the past four decades from radical mastectomy toward breast-conserving techniques such as oncoplastic lumpectomy, nipple sparing mastectomy (NSM), and the sentinel lymph node (SLN) evaluations, which provide great aesthetic satisfaction and less aggression for early-stage BCs patients (2–4). NSM of BC was firstly applied in the 1990s and by now has become an acceptable method among several breast-conserving techniques. The clinical requirements toward an even less invasive approach compared to the standard breast-conserving surgery have promoted studies investigating image-guided percutaneous ablation treatment of BC (5). As the application in other solid tumors, numerous potential ablation approaches including radiofrequency ablation (RFA), cryoablation, laser ablation, high-intensity focused ultrasound (US) and microwave ablation (MWA) have been tried in BC treatment since 1994 (6–10). There are many reasons for ablation treatment of BC, including lower cost, less morbidity, less hospitalization, better cosmetic results, and appropriate option for elderly patients with comorbidities that led to their unfit for surgery.

MWA is an exciting advance among thermal ablation techniques and has been widely used for the treatment of liver cancer (11, 12). Compared with other thermal ablation techniques, the potential advantages of MWA include larger ablation area and higher intratumoral temperatures produced by active heating (13). However, only two literatures have been published on MWA of BC patients with the initially satisfactory results since 2012 (10, 14).

MWA and NSM share the common advantage of nipple and areola sparing, and they are technically feasible for small to moderate size BC if the tumor with no clinical nipple or skin involvement (15). However, ablation is more controversial for less of evidence-based medical research for decision-making. Especially for MWA with the advantage of high thermal efficacy and potentially strong deactivation for tumor compared with other thermal ablation methods, its clinical effectiveness is to be investigated urgently. Therefore, we performed this cohort study to investigate the efficacy of ultrasound-guided percutaneous MWA without surgery as a local treatment for BC and to comparatively assess the preliminary results of MWA and NSM for treating BC.

## Materials and Methods

### Study Design and Participants

The electronic clinical records system of our hospital was checked to collect all consecutive patients who underwent percutaneous MWA or NSM for BC between October, 2014 and May, 2020. This retrospective study was approved by the institutional review board of our hospital. All the patients provided written informed consent for treatment and the informed consent for data for publication was waived by the review board as no individual information would be explored. Data were monitored by two clinicians (HW and TL, with three and five years of experience in ablation, respectively) and a statistician (Y-CL, with three years of statistical experience).

Inclusion criteria for both two groups were women, with invasive ductal carcinoma with histologic confirmation, with tumor size ≤5cm, the distance from the tumor to the nipple ≥2cm, and with no direct tumor involvement of the nipple, areola, skin, and pectoralis on imaging or physical examination, and no extensive vascular carcinoma thrombus. In addition, for the NSM group, with no significant ptosis was required, and for the MWA group the tumor was clearly visible on US was required.

Exclusion criteria were patients who were pregnant or breastfeeding, imaging suspicion of multifocality or extensive intraductal carcinoma, and previous surgery, neoadjuvant or radiation therapy of the ipsilateral breast. All the patients in the MWA group were unresectable patients for comorbidities or patients who refused surgery. Patients who met all inclusion criteria and none of the exclusion criteria were enrolled in the study.

### Pre-Procedure Evaluation

The pre-procedure evaluation and other research details are given in **Appendix E1**. Prior to the procedure the number and location of BC masses were evaluated by combination of conventional US, contrast enhanced US (CEUS), and magnetic resonance image (CEMRI). The data were analyzed by doctors JY (10-year experience in interventional radiology) and Z-yH (15-year experience in interventional radiology). Core needle biopsy was performed prior to the MWA to evaluate the pathological diagnosis of the BC and all the suspicious SLN and axillary lymph node (ALN) (based on CEUS and CEMRI) under US guidance.

### Treatments

The treatment decision was made in consensus by a team of experienced radiologists, surgeons, and oncologists in BC. The patients who were poor surgical candidates or whose preference was minimal invasion would be arranged for MWA. NSM was performed under general anesthesia as previously described for patients who elected to undergo this procedure (4, 16). Following NSM, six patients chose no reconstruction, and 37 patients were performed 1-stage tissue expander placement and 2-stage implant reconstruction.

### US-Guided MWA

US guidance was performed with a GE LOGIQ E9 scanner (GE Medical Systems US & Primary Care Diagnostics, Wauwatosa, USA) with 9.0 MHz Convex array transducer. The microwave unit (KY-2000, Kangyou Medical, Nanjing, China) is capable of producing 100 W of power at 2,450 MHz. The MWA procedure under local anesthesia and other study details are given in **Appendix E2**.

### Management of Lymph Nodes

For NSM, SLN biopsy and/or ALN dissection were performed as well as the NSM incision. ALN dissection was based on the intraoperative frozen section examination of SLN. For MWA, all the histological positive SLNs and ALNs by biopsy were ablated with moving shot technique.

### Follow-Up and Imaging Evaluation

For the MWA group, within 1–3 days after the procedure, conventional US, CEUS, and CEMRI were performed to evaluate the treatment efficacy. For both groups, conventional US was repeated for its convenience and cheapness to monitor breast at 3-month intervals during the first year after MWA or NSM and then at 6-month intervals thereafter. CEUS/MRI was performed for breast at 6-month intervals, and when US with suspicious breast lesions after MWA or NSM during follow-up (CEMRI was preferred for its accuracy in breast diagnosis, and CEUS was performed for the patients without MRI indications). Brain MRI/computed tomography, lung computed tomography, and emission computed tomography were performed for patients to evaluate systematic metastasis.

The definition of technical success and effectiveness was in **Appendix E3**. The cosmetic result was categorized as bad, moderate, good, or very good. The prespecified primary outcome measure was tumor progression (including LTP, ipsilateral breast recurrence, and systematic metastasis) evaluated according to biopsy and histological results. Prespecified secondary outcome measures were cancer specified survival, overall survival, cosmetic results, ablation volume reduction, and postoperative complications associated with the procedure and treatment.

## Statistical Analysis

The two treatment modalities were compared for patient and tumor characteristics, treatment parameters, the risk of breast recurrence, distant metastasis, and survival. Differences for categorical variables between groups were analyzed with the chi-square test or Fisher test when the assumption of number of cases per cell in the contingency tables, multiplied by two, is not fulfilled and the Student t test or non-parametric Wilcoxon rank sum test for continuous variables. Multiple clinical variables were evaluated for their potential association with tumor progression using a Cox proportional hazards regression model in univariable and multivariable analyses. We excluded each non-significant parameter with a P-value > 0.05 to finally obtain significantly independent factors. Survival and tumor progression were analyzed using the Kaplan–Meier method and compared using the log-rank test. Statistical analysis was performed using Empower(R) (www.empowerstats.com, X&Y Solutions, Inc. Boston MA). All tests were two sided, with P < 0.05 considered statistically significant.

## Results

### Patients

**Figure 1:**
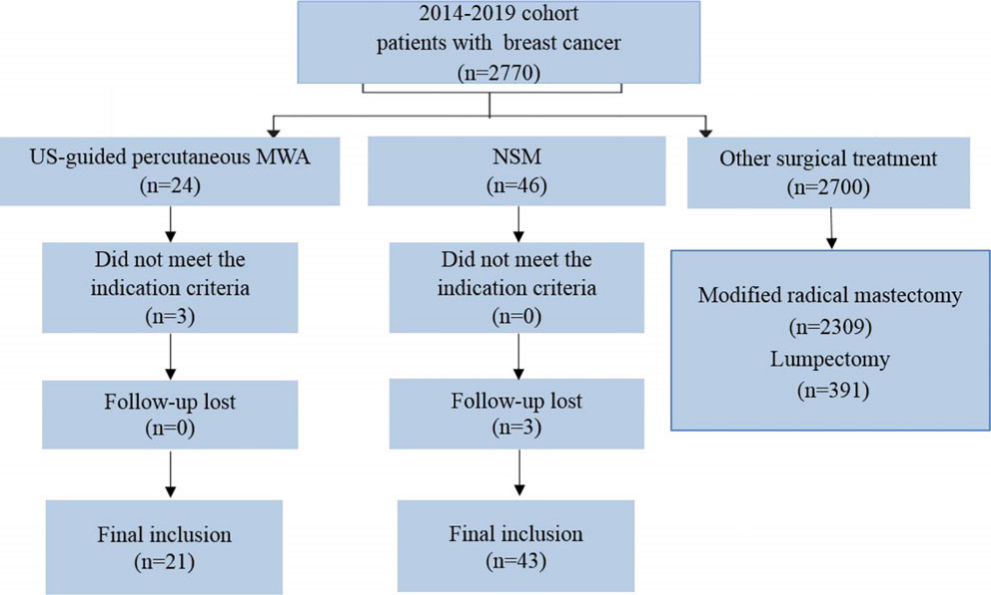
Flow of study inclusion. A total of 2,770 patients were examined with breast cancer, and 64 patients with ≤5 cm tumors treated with US-guided percutaneous MWA or NSM were finally included. US, ultrasound; MWA, microwave ablation; NSM, nipple sparing mastectomy.

During the study period, 2,770 patients with BC were assessed for eligibility for this study (**Figure 1**). Among them, 64 were enrolled in the study (21 in the MWA group and 43 in the NSM group) and no patient was lost to follow-up. The overall mean age was 47.8 years (range, 22–90 years) for the overall patients, but the mean age of the MWA group was 24 years older than that of the NSM group. Baseline characteristics of the patients are presented in **Table 1**. There was a good consistence for the tumor size, number, location, and histological type among the baseline data, but inconsistence remained for age, menopausal status, and Charlson comorbidity index (17). The MWA group had significantly more elderly patients with more comorbidities.

**Table 1:** Baseline characteristics for patients in the study group.

Parameter	MWA	NSM	*P* value
Patients (n)	21	43	
Age (yr)	64.8 ± 16.0(33–90)	39.4 ± 7.5(22–55)	<0.001
Menopausal status (Y/N)	Mar-18	42/1	<0.001
Charlson comorbidity index	3.5(0–11)	0.1 (0–2)	<0.001
Mean max size (cm)	2.4 ± 1.3(0.9–5.0)	2.3 ± 1.2(0.3–5.0)	0.81
<2.0 cm	7 (33.3%)	18 (41.9%)	0.81
2.1–3.0 cm	9 (42.9%)	13 (30.2%)	
3.1–4.0 cm	3 (14.3%)	8 (18.6%)	
4.1–5.0 cm	2 (9.5%)	4 (9.3%)	
Tumor Number (%)			0.27
1	20 (95.2%)	37 (86.0%)	
2	1 (4.8%)	6 (14.0%)	
Tumor Location (%)			0.42
Left	9 (42.9%)	23 (53.5%)	
Right	12 (57.1%)	20 (46.5%)	
TNM Stage			0.32
T1N0M0	4 (19.0%)	12 (27.9%)	0.44
T1N1M0	6 (28.8%)	5 (11.6%)	0.09
T1N2M0	0 (0.0%)	2 (4.7%)	0.32
T2N0M0	9 (42.9%)	16 (37.2%)	0.66
T2N1M0	0 (0.0%)	4 (9.3%)	0.15
T2N2M0	2 (9.5%)	4 (9.3%)	0.98
Subrogate molecular subtype*			0.04
Luminal A	9 (42.9%)	7 (16.3%)	0.02
Luminal B			
HER2 negative	4 (19.0%)	24 (55.8%)	0.007
HER2 positive	3 (14.3%)	6 (14.0%)	1
HER2 enriched (nonluminal)	0 (0.0%)	2 (4.7%)	0.31
Triple negative	2 (9.5%)	2 (4.7%)	0.46
Undefined	3 (14.3%)	2 (4.7%)	0.19

Except where indicated, data are numbers of participants, with percentages in parentheses.

MWA, microwave ablation; NSM, nipple sparing mastectomy.

*Luminal A: estrogen receptor (ER) and progesterone receptor (PR) positive, Ki67 level <20%, and human epidermal growth factor receptor type 2 (HER2) negative.

Luminal B (HER2 negative): ER positive and HER2 negative (PR < 20% or Ki67 ≥ 20%).

Luminal B (HER2 positive): ER and HER2 positive (PR < 20% or Ki67 ≥ 20%).

HER2 enriched (nonluminal): ER and PR negative and HER2 positive.

Triple negative: ER, PR, and HER2 negative.

### Treatments

In the MWA group, 21 patients with 22 tumors received 25 session treatments. Nineteen nodules were successfully treated in one MWA session, and three nodules were in two sessions. Eighteen patients were treated by MWA for advanced age or poor surgical candidates with comorbidities and three patients for preference to minimal invasion. All the patients in the NSM group underwent one operation. Four patients were performed ablation of 18 SLN/ALNs. 43 patients in the NSM group were performed SLN biopsy and 10 patients were performed ALN dissection (total 43 positive ALN) (**Table 3**). After MWA and NSM, local radioactive and systemic adjuvant treatments were performed and described in **Table 2**. The MWA group had significantly less patients receiving adjuvant systemic treatment (P = 0.03) for intolerance.

**Table 2:** Adjuvant treatment.

Parameter	MWA (*n* = 21)	NSM (*n* = 43)	*P* value
Adjuvant systemic therapy	7 (33.3%)	31 (72.1%)	0.03
Only endocrine therapy	2 (9.5%)	9 (20.9%)	0.33
Only chemotherapy	3 (14.3%)	9 (20.9%)	0.49
Endocrine therapy +Chemotherapy	2 (9.5%)	13 (30.2%)	0.008
Adjuvant radiation therapy	3 (14.3%)	7 (16.3%)	0.31
Only lymph node irradiation	1 (4.8%)	0 (0.0%)	0.15
Breast+ lymph node irradiation	2 (9.5%)	7 (16.3%)	0.47

Numbers are numbers of participants, with percentages in parentheses.

MWA, microwave ablation; NSM, nipple sparing mastectomy.

**Table 3:** Postoperative outcomes and follow-up.

Parameter	MWA (*n* = 21)	NSM (*n* = 43)	*P* value
Postoperative hospitalization time (days)	2 (1–5)	4 (2–18)	<0.001
Operative time (min)	29.9 (23.7–69.2)	130 (53–275)	<0.001
Estimated blood loss (ml)	2.0 ± 0.5	139.0 ± 100.0(20–600)	<0.001
Fever >38> (%)	0 (0.0%)	1 (2.4%)	0.48
Major complication (%)	0 (0.0%)	0 (0.0%)	>0.99
Follow-up (mons)	15.7 (5.0–47.1)	19 (4.6–58.5)	0.51
All cause death (%)	2 (9.6%)	0 (0.0%)	0.197
BC related death (%)	0 (0.0%)	0 (0.0%)	>0.99
LTP (%)	1 (4.8%)	0 (0.0%)	0.15
Isiplateral breast recurrence (%)	1 (4.8%)	1 (2.3%)	0.16
Systemic metastasis (%)	0 (0.0%)	1 (2.4%)	0.48
Costs (RMB)	25,223.5 (17,663.7–41,722.1)	22,586.5 (13,285.7–37,297.3)	0.23
Number of ablated/resected lymph nodes	0.9 (0–7)	1.3 (0–12)	0.51
Cosmetic satisfaction			<0.001
Bad (%)	0 (0.0%)	1 (2.4%)	
Moderate (%)	0 (0.0%)	10 (23.8%)	
Good (%)	0 (0.0%)	30 (71.4%)	
Very good (%)	21 (100.0%)	1 (2.4%)	

MWA, microwave ablation; NSM, nipple sparing mastectomy.

**Figure 2:**
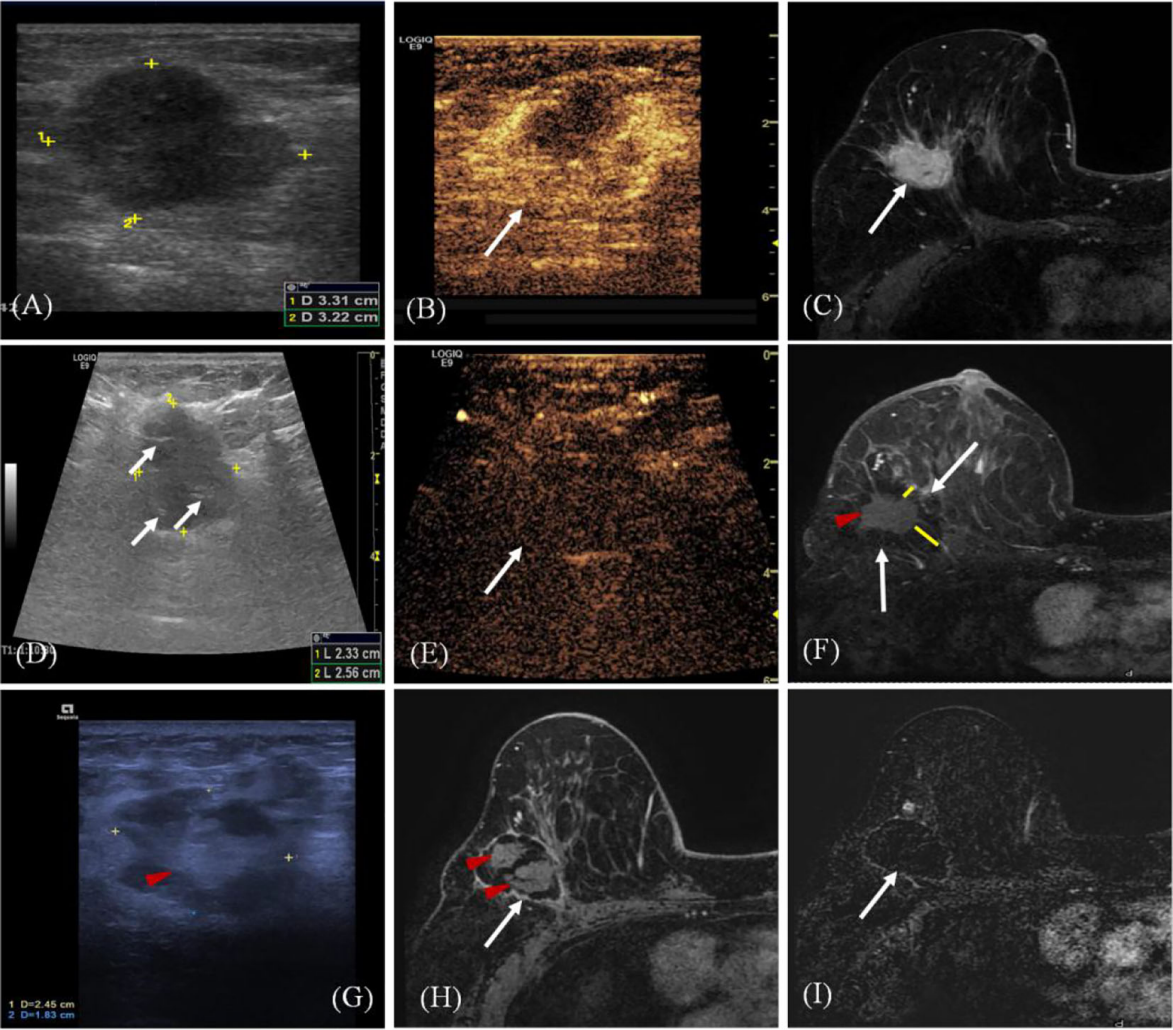
A 68-year old woman with invasive ductal carcinoma of the right breast. **(A)** Ultrasound (US) scan before microwave ablation (MWA) shows the hypoechoic mass (arrow) with size of 3.3 cm × 3.2 cm. **(B)** Contrast-enhanced US before MWA shows the mass is hyper-enhanced (arrow) in arterial phase. **(C)** Transverse contrast-enhanced magnetic resonance imaging (MRI) shows hyperintensity masses (arrow) before MWA in arterial phase. **(D)** US scan shows the heterogeneously hypoechoic mass (marker) with size of 2.6 cm × 2.3 cm immediately after MWA (ghost size). Hyperechoic needle tracts can be seen in the ablated mass (arrow). **(E)** Contrast-enhanced US immediately after MWA shows the mass is non-enhanced (arrow) in arterial phase. (F) Contrast-enhanced MRI image shows hyperintensity ghost of mass (red arrow) and the peripheral hypointensity treatment zone (white arrow) in arterial phase three days after MWA. The ablation margin is from 1.2 to 2.2 cm (yellow lines) which can be measured in the hospital information system. **(G)** US scan shows the heterogeneously ablation zone (marker) shrinks to the size of 2.5 cm × 1.8 cm at 18 months after MWA. Ghost of mass (arrow) is surrounded by hypoechoic adipose tissue. **(H)** Contrast-enhanced MRI image shows treatment zone (white arrow) is non-enhanced with clear capsule and the central hyperintensity ghost of mass (red arrow) in arterial phase at 18 months after MWA. **(I)** MRI silhouette shows no signal for the ablation zone with clear fibrous capsule and margin (arrow).

All the patients achieved technical success, and the efficacy was evaluated by CEMRI/US (**Figure 2**). The operative time of the NSM group was significantly longer than that of the MWA group (P < 0.001). Estimated blood loss was more in the NSM group (P < 0.001), but no subjects in both groups needed blood transfusion treatment (**Table 3**).

### Recurrence and Survival

The median follow-up was 26.7 months (range, 14.6–62.5 months) for overall patients. Two patients were performed core needle biopsy for their MRI results showed suspicious tumor progression around the ablation zone but achieving negative pathological diagnosis. Totally, tumor progression occurred in five patients for the two groups, which achieved consistency between MRI and pathology. Among the 51 patients with MRI and follow-up assessment, the negative predictive value (NPV), specificity and sensitivity of MRI was 100% (95% confidence interval [CI], 88.7–96.1%), 95.8% (95% CI, 86–98.8%), and 100% (95% CI, 43.9–100%).

**Figure 3:**
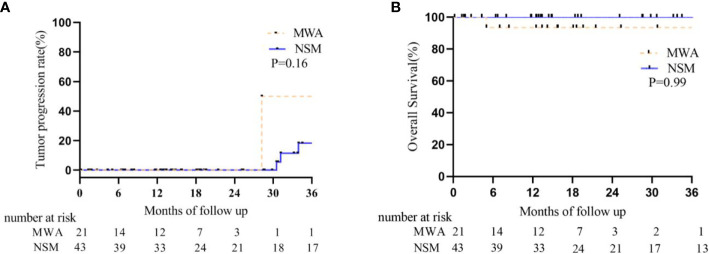
Kaplan–Meier estimates for tumor progression and survival between BC patients who underwent MWA and NSM. **(A)** Tumor progression rate. The 1-, 2-, and 3-year intra- and extra-breast recurrence rate were 0, 0, 50% in the MWA group and 0, 0, 18.3% in the NSM group, respectively (P = 0.08). **(B)** Cumulative overall survival rate. The 1-, 2-, and 3-year overall survival rate were 93.3, 93.3, 93.3% in the MWA group and 100, 100, 100% in the NSM group, respectively (P = 0.99).

One patient was diagnosed as LTP (1/22, 4.5%) at 42 months after MWA. She refused detection of tumor molecular subtype and all adjuvant treatments for 94 years old. Then she died from pulmonary heart disease at 47 months after MWA. Another 78-year old patient with molecular subtype of triple negative was diagnosed with ipsilateral breast recurrence at 28 months after MWA. She didn’t receive any treatment for fracture. No patient was diagnosed with contralateral breast or systemic recurrence in the MWA group during the follow-up. Another patient (81Y) died from cardiac arrest at 9 months after MWA. All the patients were alive in the NSM group, but one patient had ipsilateral breast recurrence at 31.2 months after NSM and was treated with mastectomy. Two patients had bone metastasis at 34 and 4 months after NSM and were controlled stably by chemotherapy of docetaxel plus cyclophosphamide. They both had the molecular subtype of Luminal B. There was no difference in tumor progression and overall survival between the two groups (**Table 3**, **Figure 3**). There was no ALN and SLN progression in all the patients.

### Univariate and Multivariable Analysis for Tumor Progression and Survival

On univariable analysis, age (hazard ratio (HR): 1.0; 95% CI: 1.0, 1.1; P = 0.05), CCI (HR: 1.7; 95% CI: 1.1, 2.6; P = 0.03), therapy method (HR: 17.3; 95% CI: 1.5, 204.3; P = 0.02), and menopausal (HR: 17.3; 95% CI: 1.5, 204.3; P = 0.02) demonstrated an association with tumor progression (**Table 4**). CCI (HR: 1.5; 95% CI: 1.0, 2.4; P = 0.04) demonstrated an association with overall survival (**Table S1**). However, we couldn’t find an independent predictor of tumor progression or survival in multivariable analyses (**Table 4**, **Tables S1**, **S2**).

**Table 4:** Univariable and multivariable analyses of predictors of tumor progression after treatment.

	Univariable		Multivariable	
Parameter	HR (95%CI)	*P* Value	HR (95%CI)	*P* Value
Age (yr)	1.0 (1.0, 1.1)	0.05	0.9(0.6,1.4)	0.69
Tumor size (cm)	0.8 (0.3, 2.1)	0.67	1.0 (0.1, 9.0)	0.99
Comorbidity Index	1.7(1.1, 2.6)	0.03	1.5(0.4,5.4)	0.49
Therapy method				
NSM	1		1	
MWA	17.3 (1.5, 204.3)	0.02	1.0 (1.0, 1.0)	NA
Menopausal				
No	1		1	
Yes	17.3 (1.5, 204.3)	0.02	81.0(0.0, inf.)	0.68
Postoperative Chemotherapy				
No	1			
Yes	1.8 (0.1, 30.8)	0.69		
Postoperative Radiotherapy				
No	1			
Yes	0.0 (0.0, inf)	0.99	.	
Postoperative Endocrinotherapy				
No	1			
Yes	0.0 (0.0, inf)	0.99		

### Volume Reduction of Ablation Zone

**Figure 4:**
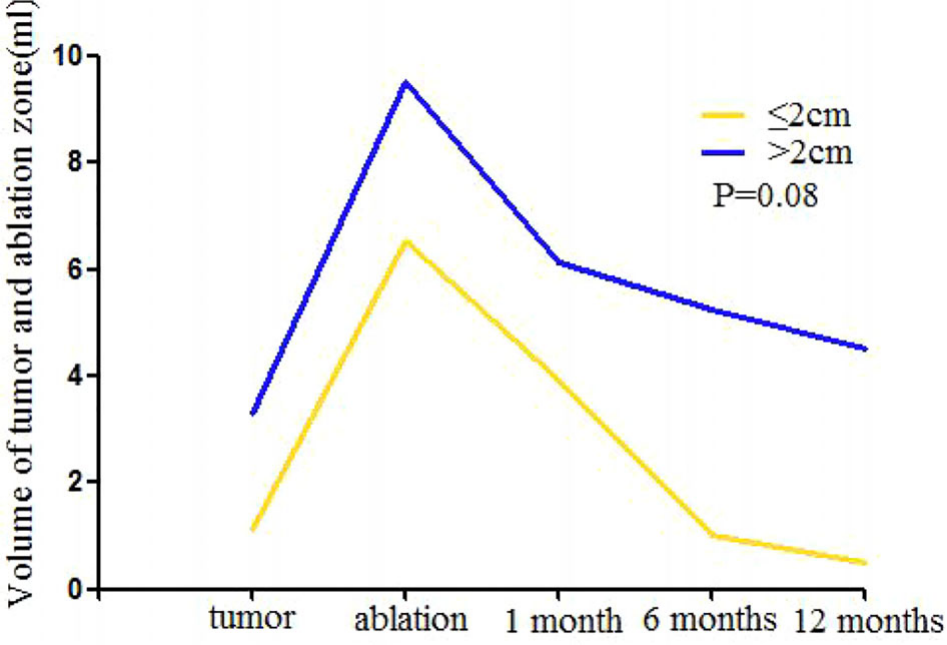
Mean volume of mass ≤ 2.0cm and >2.0cm at baseline (time of MWA) and at follow-up after treatment. One month after MWA the increased volume shows the enlarged ablation zone compared with index mass. The ablation area will shrink significantly during the 6 months after MWA and then reach stability gradually for both groups. After 6 months, the volume of mass ≤ 2.0 cm reached the level of before MWA, the volume of mass >2.0 cm was larger than the index mass continuously. There is no significant difference between two groups in volume reduction after MWA (P = 0.08).

Because some ablated tumor ghosts were not clear in the US and MRI image after one month of MWA, we calculated the volume of tumor before MWA and ablation zone by using CEMRI or CEUS if MRI was not feasible (**Appendix E4**). Compared with that of one day after MWA, the 1, 6, and 12-month median volume reduction rate of ablation zone was 35% (0–56%), 56% (0–95%), and 67 (0–97%). Volume of ablation zone showed a rapid reduction during the first 6 months after MWA and then reached stability (**Table 5**, **Figure 4**).

**Table 5:** Volume change of tumor and ablation zone.

	Tumor volume (ml)	volume of ablation zone (ml)	*P* value (compared with baseline)	*P* value (compared with immediately)	*P* value (compared with 1 month)	P value (compared with 6 months)
All tumors						
Baseline	2.1 (0.4–13.0)					
Immediately after MWA	NA	7.8 (0.5–64.7)	0.001			
1 month after MWA	NA	4.8 (0.5–51.6)	0.01	0.03		
6 months after MWA	NA	2.3 (0.2–9.1)	0.59	0.005	0.05	
12 months after MWA	NA	1.7 (0.2–6.1)	0.26	0.001	0.02	0.29
Tumor ≤2cm						
Baseline	1.1 (0.4–3.0)					
Immediately after MWA	NA	6.5 (0.5–14.5)	0.008			
1 month after MWA	NA	3.9 (0.5–9.8)	0.01	0.07		
6 months after MWA	NA	1.0 (0.2–9.1)	0.47	0.02	0.06	
12 months after MWA	NA	0.5 (0.2–5.6)	0.31	0.01	0.04	0.3
Tumor>2cm						
Baseline	3.3 (1.6–13.0)					
Immediately after MWA	NA	9.5 (1.8–64.7)	0.005			
1 month after MWA	NA	6.1 (0.8–51.6)	0.01	0.04		
6 months after MWA	NA	5.2 (1.6-–8.8)	0.53	0.03	0.34	
12 months after MWA	NA	4.5 (4.2–4.8)	0.35	0.02	0.26	0.6

NA, not available for some ablated lesions were not clear in image.

### Complications and Cosmetic Satisfaction

The safety of MWA and NSM appeared very good. Treatment was well tolerated, and there were no major complications and other adverse effects in all the patients (**Appendix E4**). For MWA, 100% of the patients reported excellent cosmetic satisfaction. For NSM, two (4.7%) patients reported bad cosmetic satisfaction, and 10 (23.3%) patients with moderate cosmetic satisfaction (**Table 3**).

## Discussion

Different from previous studies mainly focusing on ablation of BC ≤2.0 cm, our study enrolled patients with BC ≤5.0 cm because 12 (12/21, 57.1%) patients with the age older than 65 years had tumors 2.0–5.0 cm, and they lost the chance of surgery for comorbidities. Although few patients received adjuvant treatments for the old age in the MWA group, tumor progression including breast recurrences and distant metastatic recurrences has no significant difference during median 26.7 months follow-up. The only LTP patient was treated as the first case with small BC (max diameter 1.1 cm) in the MWA group, so the LTP might be attributed to the problem of limited experience to enlarge the ablation zone at the beginning of the technique, and the 94-year old patient without the chance of MRI evaluation. The favorable efficacy of MWA was better cosmetic satisfaction and less invasion with local anesthesia, shorter hospitalization time and operative time, and only means 2 ml blood loss. The clinical advantage was evident when the results of the two treatment groups were analyzed in ≤5.0 cm BC patients.

The treatment for early BC has evolved significantly since the past decade. Several randomized clinical trials have clarified a similar survival outcome between breast-conserving therapy and mastectomy (18–20). NSM is deemed as an extension of breast-conserving surgery. It is considered appropriate and oncologically safe if patients are carefully selected based on the long lasting literature data (19, 21, 22). A large number of studies have investigated RFA of BC followed by surgical resection and confirmed a high occurrence of complete tumor necrosis ranging from 80 to 100% (23). However, the evaluation of curative efficacy of ablation without subsequent surgical excision is very limited, and the comparative data from ablation alone *versus* surgery are absent.

MWA is a relatively new technique for BC with advantages of higher thermal efficiency and the potential for more complete inactivation of the tumor. The preliminary results showed 95% of patients with BC <3.0 cm could achieve complete tumor coagulation after MWA confirmed by microscopic examination (14). However, apart from BC <3.0 cm, unresectable larger tumors in senile patients were to be treated urgently by less invasive techniques. Therefore, according to our knowledge, we performed the first study comparing the ablation of BC ≤5.0 cm without subsequent excision with NSM. Both techniques are nipple sparing modalities.

The evaluation for tumor necrosis depended on the CEMRI/CEUS in all the patients who underwent MWA in our study. Pathological findings by core needle biopsy were performed only if there were suspicious lesions on image. MRI is a sensitive image for diagnosis of the breast lesion (24). It has been used to predict tissue damage after BC ablation, and previous studies suggested MRI was suitable for long-term follow-up of ablation of BC with a NPV of 92.2–97.7% (25–27). MRI evaluated the efficacy of MWA with the NPV of 100% and specificity of 95.8% in our study. And CEUS provided a good auxiliary check for 13 patients without MRI indications in our study.

According to the latest report from systematic review of imaging-guided percutaneous ablation of 1,168 BCs with the mean size from 11 to 31 mm, pooled technical success was 96% (95% CI 94–97%) and pooled TE was 75% (67–81%). RFA showed the best TE of 82% (95% CI 74–88%) and followed by cryoablation, LA, and HIFU only with 49% (95% CI 26–74). And the study concluded tumor size did not influence the TE. According to another meta-analysis including 15 clinical trials to assess the effect of RFA of BC with a total of 404 patients, pooled results showed that 89% of patients achieved TE, and several studies reported the LTP ranging from 1.37 to 14.29%. Compared with other ablation techniques, MWA of BC ≤5.0 cm achieved 100% TE and 4.5% LTP in our study, which was even superior to the previous report of ablation of <3.0 cm BC. Furthermore, the present results were from MWA of BC with relatively long follow-up information.

Just as NSM, ablation is a modality with nipple and areola sparing while with less invasion. According to this pilot study, two relatively new techniques for BC achieved similar effect. MWA used the technique by combining moving shot with fixed ablation, which showed the potential to completely eradicate the tumors with safety margin >1cm. And CEMRI/CEUS was the key image to improve the effect of MWA. Three nodules achieved complete ablation in the second session by MRI evaluation with residual tumor after the first MWA session, and totally 18 malignant lymph nodes in four patients were successfully ablated under CEUS and MRI evaluation. Certainly, this led to the slightly higher cost of MWA than that of NSM.

## Limitation

Our study has some limitations. First, we used a cohort approach, and this was a single center retrospective study with only limited participating patients and follow-up, which might lead to bias of results. Second, the margin status and tumor cell viability after MWA were not evaluated by surgery. We performed CEMRI and CEUS to improve diagnosis accuracy, which need to accumulate the experience for future ablation evaluation without surgery. Third, we only performed percutaneous biopsy for suspicious SLN and ALN on image because of patients’ refusal for surgery, which might potentially lead to positive lymph nodes missing. In conclusion, US guided percutaneous MWA and NSM seem to provide similar results for BC, with a favorable success rate and low risk of major complications. MWA could be considered as an alternative minimal-invasive treatment in early stage BC and in the elderly cases considered unfit for surgery. However, this warrants further investigation.

## Data Availability Statement

All datasets presented in this study are included in the article/**Supplementary Material**.

## Ethics Statement

The studies involving human participants were reviewed and approved by Chinese PLA General Hospital. The patients/participants provided their written informed consent to participate in this study.

## Author Contributions

JY, PL and J-dW have full access to all of the data in the study and take responsibility for the integrity of the data and the accuracy of the data analysis. PL, JY, and J-dW contributed to the conception and design of the study. PL, JY, Z-yH, TL, HW, and JJ finished the acquisition of data. JY, Y-cL, and W-zF performed the statistical analysis and interpretation. JY wrote the draft of the manuscript. PL and J-dW finished the critical revision of the manuscript for important intellectual content. All authors contributed to the article and approved the submitted version.

## Conflict of Interest

The authors declare that the research was conducted in the absence of any commercial or financial relationships that could be construed as a potential conflict of interest.

## Abbreviations

MWA, microwave ablation; NSM, nipple sparing mastectomy; BC, breast cancer; US, ultrasound; SLN, sentinel lymph node; RFA, radiofrequency ablation; LTP, local tumor progression; TE, technique effectiveness; CEUS, contrast enhanced US; CEMRI, contrast enhanced magnetic resonance image; ALN, axillary lymph node; NPV, negative predictive value; CCI, Charlson comorbidity index.
