# Supplementary material for: Microwave Ablation Versus Nipple Sparing Mastectomy for Breast Cancer ≤5 cm: A Pilot Cohort Study
Source: Front Oncol. 2020 Oct 7;10:546883. doi: 10.3389/fonc.2020.546883 (PMC7576845; doi:10.3389/fonc.2020.546883)
Supplement: Supplementary file 1 [file DataSheet_1.pdf]

Supplementary Material

1 Supplementary Data

Appendix E1. Details of pre-procedure evaluation

Information for each patient included demographics; longest diameters of tumors; tumor numbers; tumor pathological type and immunohistochemical results. Ablation variables including session, puncture, time, and power; complications; technical success (TS), technique effectiveness (TE), local tumor progress (LTP), metastasis, survival, volume reduction, and cosmetic satisfying outcomes were also measured and recorded.

1.Tumor measurement.

For MWA group, the maximum diameter of masses was measured by using CEUS and CEMRI. For NSM group, the maximum diameter of masses was measured at pathological test.

2.Biopsy

Two or three separate punctures were performed. If evidence of metastasis was detected in the SLN, the patient would be recommended to surgery. If the patient had no surgical indication or refused surgery, the metastatic SLN would be ablated simultaneously with breast tumor. Biopsy was performed using an automatic biopsy gun with an 18 G cutting needle (Bard, Phoenix, USA).

3.Imaging

US and CEUS with the contrast agent of Sonovue (Bracco Company, Milan, Italy) were performed in all patients using a GE LOGIQ E9 scanner (GE Medical Systems Ultrasound & Primary Care Diagnostics, Wauwatosa, WI, USA) with a 6.0-15.0 MHz matrix linear array multi-frequency transducer. CEMRI was performed using a 3.0-T system (Signa Echo-Speed, GE Medical Systems, Milwaukee, WI, USA) with the contrast agent of gadopentetate dimeglumine (Magnevist; Bayer Schering Pharma, Berlin, Germany).

4.Evaluation of ablation effect

In the first 3 patients before this study, the viability of the ablated tumor tissues were examined with hematoxylin and eosin (H&E) staining and/or nicotine-amide adenine dinucleotide-diaphorase (NADH-diaphorase) staining after MWA followed by mastectomy. In the first 3 patients in the study, the viability of the ablated tumor tissue was examined one month after MWA both with biopsy and CEMRI/CEUS. And for their follow-up and other 18 ablation patients, MRI was the preferred check method. Biopsy was performed only if image had suspicion.

Appendix E2. Treatment

1.Local anesthesia was performed with mixture of 2% lidocaine and 1% ropivacaine (1:1) subcutaneously and around the mass.

2.The needle antenna has a diameter of 1.6 mm (16G) and a length of 10 cm. The active tip length of MWA antenna is 3 or 5 mm.

3.After local anesthesia, the antenna was percutaneously inserted into the peripheral normal breast parenchyma and the tumor along the long axis of tumors to emit the microwave and ablated the tumor from the deep to superficial layer slowly but continuously. Pull back technique combined with fixed ablation with only one antenna was used for each patient. The applicator tip was pulled back when a heat-generated hyperechoic area was detected along the needle until the tip arrived at the 1cm margin of mass. The applicator tip was then superficially re-positioned and then pulled along the long axis of the tumor again until the hyperechoic area completely encompassed the entire tumor and surrounding parenchyma.

4.Hydro-dissection technique was used to auxiliarily ablate the tumors adjacent to skin, pectoralis and areola. Before MWA, a puncture needle (HAKKO, Nagano, Japan) with a diameter of 0.7 mm (22G) and a length of 7 cm was inserted to the site between the margin of tumors and skin, pectoralis or areola under US guidance and saline were infused slowly for adjacent tissue protection during the whole ablation procedure.

5.When the tumor was relatively far from the skin, the pectoralis or the areola (>2.0 mm), or the tumor was > 2.0 cm, an antenna with active tip of 5 mm was used; otherwise an antenna with active tip of 3 mm was used.

6.When the tumor was relatively far from the skin, the pectoralis or the areola (>2mm), a power output of 30 Watts and 30s for each applicator tip site were used, otherwise hydro-dissection technique was used and power output of 20 Watts and 10-20s for each site was used.

7.When the hyperechoic covered the whole lesion the ablation was terminated.

Appendix E3. Follow up and Imaging evaluation

1.If irregular peripheral nodular enhancement around the ablation zone was noted, this was thought to indicate the presence of residual tumor. Then the second session of ablation was performed for the patient.

2.Technical success (TS) was defined as the rate of patients in whom the operator was able to technically complete the therapy as the protocol; technical efficacy (TE) was defined as the rate of no residual tumor one month after the procedure.

3.The cosmetic result was categorized by the subject and clinician in accordance with American College of Surgeons and the Society of Surgical Oncology guidelines (Standard for breast conservation therapy in the management of invasive breast carcinoma) as bad, moderate, good, or very good.

4.Charlson comorbidity index was calculated by 19 underlying diseases with variously assigned weights that are combined into a composite score. The CCI was originally derived for hospitalized patients in general (internal) medicine, but revised versions have been validated in multiple patient populations. We cited the reference in the paper.

Appendix E4. Results

1. In MWA group, 12 patients didn't receive adjuvant therapies with the reasons for the early stage in one patient and for old age in 11 patients. In NSM group, five patients didn't receive adjuvant treatments for the early stage.

2. The median follow-up was 15.8 months (8.9-21.5 months) for the MWA group and 25.1 months (13.2-40.5 months) for the NSM group (P=0.03). There were 15 (15/21, 71.4%) and 36 (36/43, 83.7%) patients received MRI assessment in MWA and NSM groups, respectively. Other patients had no indications for MRI scan for metal coronary stents and they were evaluated by US and CEUS.

3. The volume of tumor and ablation zone at one day and 1-, 6- and 12-month after MWA were 2.1(0.4-13.0) ml, 7.8(0.5-64.7) ml, 4.8(0.5-51.6) ml, 2.3(0.2,9.1) ml and 1.7(0.2-6.1) ml.

4. Major complication included severe skin injury, abscess, hematoma, pneumothorax, tumor cell implantation, wound dehiscence, skin flap necrosis and nipple areola complex necrosis. Adverse effect included fat necrosis, nipple retraction, and mild skin injury. In MWA group, a mild sensation of heat, pain and local swell in the ablation site was experienced by all the patients, whereas, no one claimed the procedure to stop. No tranquilize medicines were given after ablation. These side effects disappeared the day after MWA. The major factor affecting the satisfaction results was the previous lumpectomy scar, insensate nipple, and asymmetric breast. All the patients reported the ablated BC became softening gradually and all the BC < 2.0 cm became impalpable after 12-month follow-up.

2 Supplementary Tables

Table S1. Univariate and Multivariate Analyses of Predictors of Over Survival after Treatment

	Univariable		Multivariable	
Parameter	HR (95%CI)	P Value	HR(95%CI)	P Value
Age(yr)	1.1 (0.9, 1.3)	0.18	1.1 (0.9, 1.4)	0.31
Tumor size (cm)	1.4 (0.5, 3.6)	0.49		
Comorbidity Index	1.5 (1.0, 2.4)	0.04	1.2 (0.6, 2.6)	0.58
Therapy method				
NSM	1			
MWA	inf. (0.0, Inf)	1		
Menopausal				
No	1			
Yes	inf. (0.0, Inf)	1		
Postoperative Chemotherapy				
No	1			
Yes	0.0 (0.0, Inf)	0.99		

Postoperative Radiotherapy			
No	1		
Yes	0.0(0.0, inf)	0.99	
Postoperative Endocrinotherapy			
No	1		
Yes	0.0(0.0, inf)	0.99	.

Table S2. Univariate and Multivariate Analyses of Predictors of Ipsilateral Breast Recurrence after Treatment

	Univariable		Multivariable	
Parameter	HR (95%CI)	P Value	HR（95%CI）	P Value
Age(yr)	1.1 (1.0, 1.2)	0.07	1.1 (0.9, 1.4)	1.0
Maximum diameter of tumors(cm)	0.5 (0.1, 2.2)	0.38	0.0 (0.0, Inf)	1.0
Therapy method				
NSM	1		1	
MWA	inf. (0.0, Inf)	1.0	0.0 (0.0, Inf)	1.0
Menopausal				
No	1		1	
Yes	inf. (0.0, Inf)	1.0	inf. (0.0, Inf)	1.0
Comorbidity Index	1982.9 (0.0, Inf)	1.0	56.3 (0.0, Inf)	1.0
Postoperative Chemotherapy				
No	1		1	
Yes	1.1 (0.0, 32.0)	0.96	431135.5 (431135.5, 431135.5)	<0.001
Postoperative Radiotherapy				
No	1		1	

Yes	0.0 (0.0, Inf)	0.99	11.8 (11.8, 11.8)	<0.001
Postoperative Endocrinotherapy				
No	1		1	
Yes	0.0(0.0, inf)	0.99	. 2.0 (2.0, 2.0	<0.001
Treated lymph Nodes	0.0(0.0, inf)	0.99	2.9 (2.9, 2.9)	<0.001
Clinical Staging	0.0(0.0, inf)	0.99	0.2 (0.2, 0.2)	<0.001
